# A Practice Model for Palliative Radiotherapy

**DOI:** 10.7759/cureus.83316

**Published:** 2025-05-01

**Authors:** Alina Zheng, Alec Zheng, Alan Zheng, Xiaodong Wu, Beatriz Amendola

**Affiliations:** 1 Radiation Oncology, Innovative Cancer Institute, South Miami, USA; 2 Neuroscience and Behavioral Biology, Emory University, Atlanta, USA; 3 Medical Physics, Executive Medical Physics Associates, North Miami Beach, USA

**Keywords:** cancer, hospice, pain management, palliative care, radiotherapy

## Abstract

Despite well-recognized challenges in implementing palliative radiation therapy (PRT), progress remains slow, and conventional approaches have yielded limited success. A transformative strategy is required to overcome systemic barriers and establish a sustainable PRT infrastructure. This proposal presents a novel model to improve accessibility, affordability, and integration into palliative care by addressing key obstacles in training, regulation, facility development, and treatment protocols.

A specialized certification track within radiation oncology residency programs is proposed, enabling palliative care physicians to obtain limited PRT licenses under the supervision of fully licensed radiation oncologists. Regulatory adjustments should facilitate this framework, ensuring compliance while expanding the PRT workforce. Dedicated PRT facilities-affiliated with comprehensive radiation therapy centers (CRTCs) and integrated into hospice settings-will enhance accessibility by reducing logistical and financial burdens. These facilities will utilize cost-effective infrastructure, including refurbished linear accelerators, modular construction, and remote physics and dosimetry support, ensuring operational costs remain significantly lower than those of conventional radiotherapy centers.

Optimizing PRT delivery requires shifting clinical strategies toward single-fraction treatment as the primary approach, followed by hypofractionation treatment when necessary. Systematic studies with a PRT-oriented mindset should establish PRT-specific treatment recommendations and recommendations, moving away from conventional radiation therapy protocols.

By addressing key barriers in education, regulation, infrastructure, and clinical strategy, this model offers a path toward sustainable PRT implementation. While requiring initial investment and regulatory adjustments, it has the potential to improve end-of-life care for terminally ill cancer patients, ensuring greater dignity and comfort while establishing a robust foundation for future reimbursement models.

## Introduction

Palliative radiation therapy (PRT) is essential for managing symptoms in end-stage cancer patients, providing relief from pain, tumor-related bleeding, dyspnea, and neurological deficits [1,2]. The primary aim of PRT is to enhance comfort rather than cure, ensuring better quality of life (QOL) in the final stages of illness [3]. A study showed PRT’s effects led to a downward shift on the World Health Organization (WHO) Analgesic Ladder, a part of the WHO Cancer Pain and Palliative Care Management program [4]. The WHO Ladder of pain control consists of three steps: non-opioid analgesics for mild pain, weak opioids for moderate pain, and potent opioids for severe and persistent pain. PRT decreased the need for opioids, improving QOL by reducing pain and lowering opioid requirements.

Despite its benefits, PRT remains underutilized due to disparities in access, physician preferences, and systemic barriers [5-7]. While approximately 45% of radiation therapy is done with palliative intent, only 3% of the about 40% of hospice patients with advanced cancer receive PRT [5]. Additionally, misconceptions about palliative care often prevent early symptom management [6]. Many patients receive late referrals, limiting treatment effectiveness [8]. This highlights a significant gap in care where many patients may not be receiving the full benefits of radiation therapy for symptom management.

Education is a contributing factor, with many radiation oncologists receiving limited palliative care training [2]. Although most radiation oncology program directors acknowledge the importance of palliative care education, only 67% of programs offer robust educational opportunities [9]. The primary goal of PRT is symptom relief with minimal treatment burden, and the choice of regimen must balance efficacy, side effects, logistical considerations, and patient prognosis. The selection of a PRT regimen depends heavily on the life expectancy of end-stage cancer patients. Hypofractionation and stereotactic body radiation therapy (SBRT), including single-fraction radiation therapy (SRT), have improved efficiency, yet implementation of these technologies remains inconsistent [10,11]. A study showed that among patients who received PRT within 30 days of death, 42% of them could not complete their courses [12]. According to published data, over 50% of patients with bone metastases are treated with 10 or more fractions, and among patients with non-spine bone metastases, approximately only 5% are treated with SRT [5]. While three-quarters of medical directors know that PRT is just as effective when done with a single fraction, only about 20% of administrators and nurses are aware of that same fact. This discrepancy, paired with the fact that 43% of radiation oncologists feel reluctant to perform SRT [13], contributes to the underuse of SRT in treating uncomplicated bone metastases, despite its proven effectiveness for pain management.

For optimal care in a life-limited situation, the referral process must be streamlined. Issues like delays in consultation and difficulties in contacting radiation oncologists negatively impact referrals [14,15]. While increased familiarity can help hospice and palliative medicine (HPM) workers navigate the process, processes must be streamlined to improve the efficiency with which patients move between providers. Improved connectivity and collaboration between radiation oncologists and palliative care physicians are essential [6]. To address the underutilization of PRT and the proper selection of treatment techniques for hospice patients, it is crucial for HPM programs and radiation therapy programs to better understand each other's practices.

Reimbursement for PRT is generally limited [2,5]. High-cost state-of-the-art equipment, skilled manpower, and strict regulatory requirements on the comprehensive training of radiation oncologists are positioned to elevate the quality of conventional RT. Without remarkable amounts of coordination and collaboration, the cost to the radiation oncology practice administering such treatment substantially exceeds reimbursement for the care. This is perhaps one of the greatest impediments to greater implementation of efficiency in PRT administration.

Here, we propose a new path. By tackling key obstacles - education, regulation, workforce and facility configuration, procedural optimization, and cost management - we aim to create opportunities for a sustainable PRT infrastructure.

## Technical report

To date, the challenges hindering the consistent and effective implementation of PRT have been well recognized. However, progress remains slow, and conventional approaches have yielded limited success. A more transformative and disruptive strategy is required to overcome these persistent barriers. The practice model proposed herein emerges from the fresh perspective of the first three authors, who conceived the idea during their internships in radiation therapy. This model advocates for radical changes to certain elements within the established radiation therapy infrastructure. While its feasibility requires further scrutiny, it has the potential to create new opportunities for PRT and offer hope to patients in the final stages of their battle with cancer.

A proposed new practice model for PRT

Training and Regulatory Adjustments

A special certification track within standard radiation oncology residency programs for palliative care physicians should be established, enabling them to obtain a limited radiation therapy license specifically for PRT. Such a limited license would allow palliative care physicians to administer PRT under supervision and support from fully licensed radiation oncologists. The training should focus on the essential aspects of PRT, such as patient assessment, treatment planning, and delivery, while ensuring adherence to safety and efficacy standards.

The training program should encompass a comprehensive blend of theoretical knowledge, practical skills, and interdisciplinary collaboration to ensure effective and safe delivery of PRT. Participants should gain a solid foundation in the basic principles of radiation therapy, the role of PRT in palliative care, and essential radiation safety protocols. Practical training should include hands-on experience with simulation tools, supervised clinical practice, and case studies to develop competency in delivering PRT. Additionally, the program should emphasize interdisciplinary collaboration, equipping trainees with the skills necessary to work alongside palliative care physicians with limited radiotherapy licenses and radiation oncologists in comprehensive radiation therapy facilities. This includes training in effective communication and referral processes for cases requiring more complex radiation therapy.

In conjunction, regulatory adjustments should focus on accrediting regulatory bodies to approve and oversee specialized training programs, ensuring they meet established standards. Additionally, regulations should facilitate the granting of limited radiation therapy licenses to palliative care physicians who have completed the certified training, allowing them to perform PRT within specified scopes and guidelines under the direct or indirect supervision of fully licensed radiation oncologists. Mechanisms for monitoring compliance should also be established to ensure adherence to training and practice standards, including mandatory continuing education requirements.

Business development efforts should focus on establishing partnerships between hospice and palliative medicine fellowship programs and radiation oncology residency programs to develop and deliver PRT certification tracks. Additionally, securing funding through grants and financial support from healthcare foundations, government agencies, and philanthropic organizations will be essential to subsidize training costs. Marketing initiatives should promote these certification programs through medical conferences, professional networks, and online platforms to attract participants and build awareness within the medical community.

Establishment of Dedicated PRT Facilities

Dedicated PRT facilities within or adjacent to hospice centers in densely populated cities or suburban areas should be built to streamline operations, reduce travel time for patients, and make radiation therapy an integrated part of palliative care. Each PRT facility is necessarily a direct affiliation of a comprehensive radiation therapy center (CRTC) that would assert supervision and support of the PRT operation, while the actual treatments are to be administered by the palliative care physician(s) with a PRT certificate and a special limited license. 

The facility design for PRT should include linear accelerators (Linacs) with basic configurations, which are sufficient for delivering PRT while reducing equipment costs. Additionally, the facility should prioritize a patient-centered environment by incorporating welcoming waiting areas, ensuring easy accessibility for stretcher-bound and wheelchair-bound patients, and integrating supportive care services to enhance patient comfort and overall care.

Cost management for a dedicated PRT facility should ensure that total expenses remain under one-third of those of a conventional radiotherapy center, making it a financially viable model. This can be achieved through modular construction to reduce time and costs, investment in cost-effective yet reliable radiation therapy equipment, and optimized staffing models. The availability of refurbished linacs presents a significant opportunity. The facility should employ palliative care physicians with specialized PRT training certificates, working under the direct supervision or remote support of fully licensed radiation oncologists from an affiliated CRTC. Additionally, physics and dosimetry planning can be provided remotely through the affiliated CRTC, maintaining high standards of care while minimizing operational costs.

Business development for dedicated PRT facilities should focus on conducting thorough feasibility studies to identify optimal locations based on population density, patient demographics, existing hospice services, and potential demand. Securing funding through public-private partnerships, healthcare grants, charity organizations, and investments from private equity firms interested in healthcare infrastructure is essential for financial viability. Developing sustainable operational models that balance cost efficiency with high-quality care is critical, including partnerships with existing hospice centers and CRTCs to share resources and reduce overhead costs. Additionally, marketing and outreach efforts should raise awareness among healthcare providers, patients, and the community through targeted campaigns that highlight the benefits of dedicated PRT facilities, such as improved accessibility, patient-centered care, and integrated supportive services.

Integrating PRT into conventional RT centers has proven ineffective, failing both providers and patients - a hard reality. Establishing dedicated PRT facilities with substantially lower hardware and manpower costs is the first step toward building an affordable and sustainable PRT infrastructure.

Optimizing PRT Strategy

The timing of palliative RT is crucial, especially in patients with a limited prognosis. Research indicates that less than 15% of cancer patients receive palliative RT in the last month of life. However, a significant proportion of these patients may not complete their treatment due to rapid health decline, leading to minimal benefit [16]. Studies have also found that clinicians often overestimate patient survival, leading to longer and more burdensome RT regimens that patients may not live to complete. In one study, about half of the patients spent more than 60% of their remaining lifespan undergoing treatment [10].

Given the relatively shorter life expectancy, a single-fraction approach should be considered first, followed by a hypofractionation scheme of fewer than five fractions. The fractional dose should be sufficiently high to ensure rapid symptom relief while maintaining tolerable levels of acute radiation toxicity. Currently, dose prescriptions are still largely based on experience with conventional radiation therapy. One factor hindering the adoption of SRT is the conventional mindset shaped by standard RT, where SRT and SBRT are associated with highly procedural complexities. A PRT-oriented approach, justified by significantly simplified protocols and reinforced through specialized training, can help overcome this hesitation. Additionally, systematic clinical studies to establish PRT-specific dose recommendations will be invaluable in guiding its effective implementation.

## Discussion

PRT has remarkable and demonstrated significant potential to enhance the QOL for patients with advanced cancer, particularly in hospice settings. Despite this, its integration into palliative care remains limited due to various systemic challenges, including gaps in education, logistical inefficiencies, and inadequate reimbursement mechanisms. These barriers highlight the need for a multifaceted approach to improve PRT accessibility and delivery.

Education and training represent a critical area for intervention. Expanding radiation oncology residency curricula to include palliative care and introducing specialized PRT certification programs for palliative care physicians can help address the lack of expertise in hospice settings [9]. Additionally, fostering better collaboration between radiation oncologists and hospice teams through interdisciplinary training and regular communication is essential for improving care coordination and referral processes [17].

Logistical issues, such as delays in consultation and the underutilization of single-fraction radiotherapy, further exacerbate the challenge. Simplifying referral processes, leveraging telemedicine for remote consultations, and prioritizing SRT for patients with limited life expectancy can improve treatment efficiency while minimizing the burden on patients and caregivers. Furthermore, integrating radiation oncologists into multidisciplinary teams can enhance the alignment of care strategies.

The establishment of dedicated PRT facilities affiliated with CRTCs offers a promising solution to many of these challenges, especially in densely populated areas. These facilities, equipped with basic linear accelerators and staffed by palliative care physicians with specialized PRT training, can deliver localized, cost-effective care. Remote supervision by fully licensed radiation oncologists ensures adherence to safety and quality standards. Such centers, designed to focus on operational efficiency and patient-centered care, have the potential to significantly reduce logistical and financial barriers.

Finally, addressing reimbursement challenges is imperative. Current reimbursement models often fail to cover the costs associated with efficient PRT delivery, discouraging its broader implementation. Legislative efforts, such as the Radiation Oncology Case Rate proposal [18], could provide critical support for increasing the availability and sustainability of PRT services. Ultimately, we must fully acknowledge the fundamental logistical and operational differences between conventional RT and PRT to justify the need for radical reform. Overcoming current barriers will require both effort and initial investment. While the majority of research rightly focuses on advancing conventional RT, a reasonable proportion should be dedicated to developing a simplified, cost-effective PRT model. Without such groundwork, any attempt to establish a new reimbursement structure will lack a strong foundation for meaningful change.

## Conclusions

A cost-effective, seamlessly integrated palliative care system is essential to unlocking PRT’s full potential in improving the QOL for terminally ill cancer patients. However, systemic barriers hinder this integration. Radical changes, including expanding training for palliative care providers, streamlining processes, and optimizing treatment effects while minimizing cost, are foundational steps. Establishing low-cost dedicated PRT centers near hospice facilities, staffed by specially trained palliative care physicians and supported by comprehensive radiotherapy centers, offers a transformative approach to enhancing accessibility and efficiency.

Given RT’s proven advantages over morphine and the imperative to prioritize end-stage patients' QOL, fully integrating PRT into hospice care is a necessary but challenging endeavor. While the proposed model may require a sizable initial investment, it has the potential to drive meaningful regulatory and reimbursement adjustments, ultimately establishing a sustainable PRT infrastructure. This, in turn, will help alleviate suffering and provide terminally ill cancer patients with greater dignity and comfort in their final days.
